# The RECAP Test Rapidly and Reliably Identifies Homologous Recombination-Deficient Ovarian Carcinomas

**DOI:** 10.3390/cancers12102805

**Published:** 2020-09-29

**Authors:** Lise M. van Wijk, Sylvia Vermeulen, Matty Meijers, Manuela F. van Diest, Natalja T. ter Haar, Marthe M. de Jonge, Nienke Solleveld-Westerink, Tom van Wezel, Dik C. van Gent, Judith R. Kroep, Tjalling Bosse, Katja N. Gaarenstroom, Harry Vrieling, Maaike P. G. Vreeswijk

**Affiliations:** 1 Department of Human Genetics, Leiden University Medical Center, 2300 RC Leiden, The Netherlands; L.M.van_Wijk@lumc.nl (L.M.v.W.); S.Vermeulen@lumc.nl (S.V.); matmeijers@hotmail.com (M.M.); manuelafran@live.nl (M.F.v.D.); H.Vrieling@lumc.nl (H.V.); 2 Department of Pathology, Leiden University Medical Center, 2300 RC Leiden, The Netherlands; N.T.ter_Haar@lumc.nl (N.T.t.H.); M.M.de_Jonge@lumc.nl (M.M.d.J.); N.Solleveld-Westerink@lumc.nl (N.S.-W.); T.van_Wezel@lumc.nl (T.v.W.); T.Bosse@lumc.nl (T.B.); 3 Department of Molecular Genetics, Erasmus MC, 3000 CA Rotterdam, The Netherlands; d.vangent@erasmusmc.nl; 4 Department of Medical Oncology, Leiden University Medical Center, 2300 RC Leiden, The Netherlands; J.R.Kroep@lumc.nl; 5 Department of Gynecology, Leiden University Medical Center, 2300 RC Leiden, The Netherlands; K.N.Gaarenstroom@lumc.nl

**Keywords:** epithelial ovarian carcinoma, homologous recombination deficiency, RECAP test, RAD51, BRCA1, BRCA2

## Abstract

**Simple Summary:**

The sensitivity to PARP inhibitors (PARPi) is related to tumor-specific defects in homologous recombination (HR) and extends beyond *BRCA1/2-*related deficiencies. A robust method to identify HR-deficient (HRD) carcinomas is therefore of utmost clinical importance. In this study, we evaluated the use of a functional test (the RECAP test) for the identification of HRD ovarian carcinomas. Forty-nine epithelial ovarian carcinomas (EOC) were analyzed by the RECAP test. Thirty-nine of these tumors were of the high-grade serous (HGSOC) histologic subtype. Ten out of these 39 HGSOC specimens showed HRD (26%), whereas ovarian carcinomas of other histologic subtypes (*n* = 10) were all HR-proficient (HRP). Eight out of 9 sequenced HRD tumors showed pathogenic *BRCA1/2* variants or *BRCA1* promoter hypermethylation. This study shows that the RECAP test is a reliable and rapid test to identify functional deficiencies in HR and a good alternative to DNA-based HRD tests.

**Abstract:**

Recent studies have shown that the efficacy of PARP inhibitors in epithelial ovarian carcinoma (EOC) is related to tumor-specific defects in homologous recombination (HR) and extends beyond *BRCA1/2* deficient EOC. A robust method with which to identify HR-deficient (HRD) carcinomas is therefore of utmost clinical importance. In this study, we investigated the proficiency of a functional HR assay based on the detection of RAD51 foci, the REcombination CAPacity (RECAP) test, in identifying HRD tumors in a cohort of prospectively collected epithelial ovarian carcinomas (EOCs). Of the 39 high-grade serous ovarian carcinomas (HGSOC), the RECAP test detected 26% (10/39) to be HRD, whereas ovarian carcinomas of other histologic subtypes (*n* = 10) were all HR-proficient (HRP). Of the HRD tumors that could be sequenced, 8/9 showed pathogenic *BRCA1/2* variants or *BRCA1* promoter hypermethylation, indicating that the RECAP test reliably identifies HRD, including but not limited to tumors related to *BRCA1/2* deficiency. Furthermore, we found a trend towards better overall survival (OS) of HGSOC patients with RECAP-identified HRD tumors compared to patients with HRP tumors. This study shows that the RECAP test is an attractive alternative to DNA-based HRD tests, and further development of a clinical grade RECAP test is clearly warranted.

## 1. Introduction

Surgical cytoreduction combined with platinum-based chemotherapy has traditionally been the standard of care in the treatment of epithelial ovarian carcinoma (EOC) patients [1,2]. However, a series of recent clinical trials showed that Poly (ADP-Ribose) Polymerase inhibitor (PARPi) as maintenance treatment of EOC patients with platinum-sensitive cancers results in significant improvement of both progression-free survival (PFS) and overall survival (OS) in newly diagnosed and recurrent EOC [3,4,5,6,7,8,9,10,11]. This led to FDA and EMA approval of various PARPi as maintenance therapy in patients with platinum-sensitive EOC, both in the primary and recurrent setting. Although the presence of pathogenic variants in *BRCA1* or *BRCA2* is the best genetic predictor for a good clinical response to PARPi therapy, multiple studies have shown that PARPi efficacy is also observed in patients with non-*BRCA1/2-*deficient carcinomas [4,5,9,12]. Since current evidence suggests that a deficiency in homologous recombination (HR) underlies sensitivity to PARPi, a search for biomarkers that correlate with HR Deficiency (HRD) was initiated with the aim of developing a single method that can identify all patients who could potentially benefit from PARPi treatment [13,14,15,16] (Table S1). 

Most of the currently available HRD tests are DNA-based. Using gene-specific analyses, the percentage of HRD high-grade serous ovarian carcinomas (HGSOC) is estimated to be as high as 50% [17]. In addition to the presence of pathogenic variants in *BRCA1* and *BRCA2* (20%), inactivation of other genes involved in the HR pathway (6%) and the epigenetic silencing of *BRCA1* (10%) or *RAD51C* (3%) have also been reported in HGSOC [13,17,18]. The prevalence of HRD in other histologic subtypes of EOCs such as low grade serous, endometrioid, clear cell, and mucinous OC is still unclear [19]. In addition to gene-specific analyses, more complex DNA-based approaches, such as the identification of ‘genomic scars’, are now being explored as potential biomarkers of HRD. These methods focus on identifying mutational signatures caused by the use of alternative, error-prone repair pathways to repair DNA double-strand breaks (DSBs) in the absence of HR, and include copy number-based methods incorporating loss of heterozygosity (LOH) [20,21,22,23], Telomeric Allelic Imbalances (TAI) and Large-scale State Transitions (LST) [21,23,24,25]. Similar but more complex whole genome sequencing-based approaches include HRDetect, a weighted model that includes features such as microhomology-mediated deletions, base substitution, rearrangement signatures and LOH [26]. HRD-associated genomic scars have been shown to facilitate identification of both *BRCA1/2*-deficient (m*BRCA*) as well as *BRCA1/2*-unrelated (wt*BRCA*) HRD [12,20,23,26]. 

The predictive value of DNA-based HRD status (defined by the presence of pathogenic variants in *BRCA1/2* and/or a specific genomic scar) for PARPi treatment benefit in EOC patients has been evaluated in a number of clinical studies. These studies revealed that although both *BRCA* mutation status and HRD status provided information regarding the magnitude of the potential treatment benefit of a PARPi in a given patient population, these biomarkers did not sufficiently discriminate between patients that would or would not benefit from treatment [4,5,9,12,27]. Overall, while these DNA-based tests provide valuable proof of concept for the existence of an additional group of HRD tumors beyond those related to *BRCA1/2*, DNA-based approaches also suffer from a number of important drawbacks regarding the identification of HRD tumors. Firstly, they cannot identify all EOC patients who would benefit from treatment with PARPi [4]. Secondly, the interpretation of modern sequencing data is far from straightforward, as gene-based analyses often detect variants of uncertain significance (VUS) without a clear relationship to HRD, and signature-based approaches identify a relatively large fraction (18%) of HR-intermediate cases in EOC [26]. Thirdly, DNA-based assays are relatively complex, costly, and time-consuming. 

As an alternative to DNA-based HRD tests, functional assays that assess the ability of replicating tumor cells to accumulate RAD51 protein at DNA DSBs were developed for use in breast, ovarian and endometrial cancer [28,29,30,31,32,33,34,35,36,37]. A major advantage of this approach is that RAD51-based tests detect the current HR status of the tumor, irrespective of underlying genetics. Furthermore, the “static” HRD status as measured by DNA-based approaches may overestimate the number of tumors that are “functionally” HRD because they make no account for undetected reversion mechanisms [16,38,39,40,41]. Studies describing RAD51-based tests have shown that they are able to identify HRD tumors, including but not limited to *BRCA1/2*-related tumors, and can detect phenotypic reversion of the HRD phenotype in *BRCA*-related tumors [28,29,30,31,32,33,34,35,37,41]. In EOC, impairment of HR, as assessed in primary cultures established from ascitic fluid, correlates with PARPi sensitivity both in vitro and in clinical studies [30,31]. A recent study using low passage, primary patient-derived tumor and ascites cells, confirmed the predictive power of a functional HR score for platinum sensitivity [35]. 

The REcombination CAPacity test (RECAP) test is a RAD51-based functional test which was previously successfully used for the analysis of HR function in solid breast and endometrial carcinomas [36,37]. In contrast to other RAD51-based tests, the RECAP test enables the assessment of HR on primary tumor tissue without the need to dissociate tissue and culture tumor cells, greatly reducing the time required to perform the test. 

In the current study, additional quality controls are described that enable the use of the RECAP test in both solid EOC tumors and ascites-derived EOC tumor cells, taking into account future clinical implementation in routine diagnostic pathology. We demonstrate that the RECAP test allows the rapid and reliable determination of the functional HR status of EOC, and then explore its correlation with best overall therapy response and overall survival (OS). 

## 2. Results 

### 2.1. REcombination CAPacity (RECAP) Test

**Figure 1:**
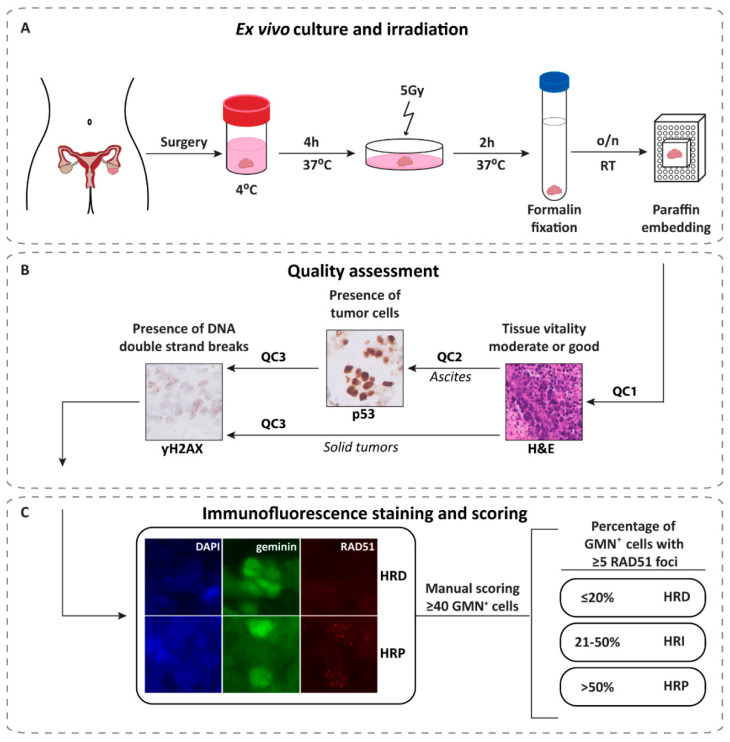
RECAP test procedure. (**A**) After surgery, tumor specimens were placed in OSE medium and incubated for 4 h at 37 °C on a 60 rpm rotating platform prior to irradiation with 5 Gy. Tumor specimens were fixed two hours after irradiation and embedded in paraffin. (**B**) H&E slides were evaluated for tissue quality (QC1) by an experienced pathologist. For ascites specimens an additional p53 IHC staining was performed to confirm the presence of tumor cells (QC2). All tumor specimens were evaluated for the presence of DNA double strand breaks based on yH2AX IHC (QC3). (**C**) A RAD51/GMN IF was performed on tumor specimens that passed the quality assessment. The RECAP score indicates the percentage of GMN^+^ cells (≥40 cells) with RAD51 foci (≥5 foci). The whole procedure can be performed on multiple specimens simultaneously. Abbreviations: o/n = overnight; RT = room temperature; QC = quality control; H&E = Hematoxylin and Eosin; IF = immunofluorescence; GMN^+^ = geminin-positive; HRP = HR-Proficient; HRI = HR-Intermediate; HRD = HR-Deficient.

The RECAP test has been described in detail in earlier publications [35,36,37], but in this study, we adapted the protocol to make it suitable for direct use with solid tumor and ascites specimens, removing the need for tumor dissociation and cell culture procedures (Figure 1). The RECAP test can include up to 25 tumor specimens simultaneously, and works equally well on fresh or thawed cryopreserved specimens (Figure S1). The use of cryopreserved samples greatly increases the clinical utility of the RECAP test, allowing one e.g., to collect tumor tissues from different centers and perform the test in one laboratory. The entire procedure (quality assessment and RECAP test) for a capacity of 25 samples can be completed within two weeks and represents a 25-h hands-on workload for one person (Table S2). The cost price of the RECAP test is more than 10-fold lower compared to DNA-based alternatives (Table S3) and can be completed in less time than a *BRCA* NGS gene panel (Table S2).

### 2.2. Identification of Homologous Recombination-Deficient Tumors Using the RECAP Test 

In total, 50 (33 solid and 17 ascites) tumor specimens (71% of the collected specimens) met the quality criteria and were therefore eligible for the RECAP test (Figure S2A, Table S4). Of the 50 specimens analyzed, RECAP scores could be determined for 49 tumor specimens (98%) obtained from 48 patients (33 solid tumors and 16 ascites specimens; hereafter referred to as ‘RECAP specimens’), as one ascites specimen had to be excluded due to an insufficient number of GMN^+^ cells (<40) (Figure S2B, Table S4). Two tumor specimens were obtained from the same patient (case 18 and 28): one at primary cytoreductive surgery (solid tumor) and one at recurrence (ascites). 

**Figure 2:**
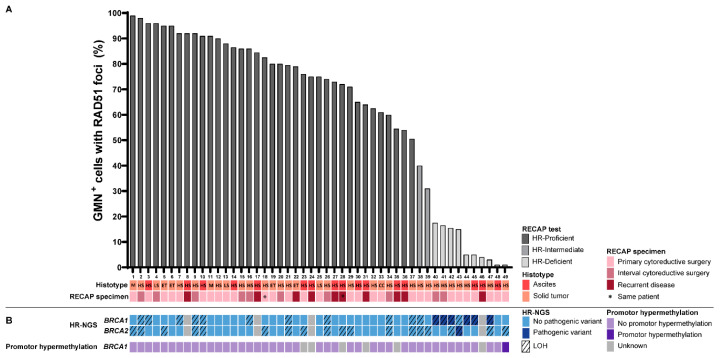
RECAP scores of 49 EOC tumor specimens. (**A**) RECAP scores were calculated as the average percentage of GMN^+^ cells with ≥5 RAD51 foci scored by two independent observers, and specimens were classified as HRP (51–100%), HRI (21–50%) or HRD (0–20%). (**B**) NGS analysis identified seven HRD tumors harboring a pathogenic variant in *BRCA1* or *BRCA2* with concomitant LOH. No pathogenic variants in *BRCA1* or *BRCA2* were identified in HRI and HRP tumors. *BRCA1* promoter hypermethylation was observed in case 49. Pathogenic variants in other HR genes were tested in the HRD and HRI tumors, but none were identified. Abbreviations: HR = Homologous Recombination; HRP = HR-Proficient; HRI = HR-Intermediate; HRD = HR-Deficient; RECAP = REcombination CAPacity; GMN^+^ = geminin-positive; NGS = next-generation sequencing; HS = high-grade serous; LS = low-grade serous; M = mucinous; CC = clear cell; ET = endometrioid; LOH = loss of heterozygosity.

Based on the RECAP test, ten (20%) of the 49 tumor specimens were found to be HRD (i.e., a RECAP score 0–20%), 37 (76%) were HRP (i.e., a RECAP score 51–100%) and two tumors (4%) were HRI (i.e., a RECAP score 21–50%). Examples of immunofluorescent stained slides are presented in Figure S3. Median RECAP scores differed by 6% (range: 0–34%) between two independent observers, with a high interrogator reliability for final HR group assignment (κ = 0.71). Thirty-nine of the 49 tumors (80%) were HGSOC, and of these tumors 26% (10/39) were HRD (Figure 2A). The remaining ten tumors (20%) were of other histologic subtypes and were all HRP, thus HRD was restricted to HGSOC in this set of tumors.

### 2.3. Genetic Alterations in HR Genes

In an effort to determine the molecular basis for loss of HR functionality in tumors displaying HRD, (epi)genetic analyses were performed in nine HRD tumors. One HRD tumor could not be analyzed due to an insufficient amount of tumor DNA. Pathogenic variants in *BRCA1*, *BRCA2*, or *BRCA1* promoter hypermethylation were detected in eight out of nine (89%) HRD tumors in this particular cohort. Six HRD tumors harbored pathogenic variants in *BRCA1* with concomitant LOH of the wild-type allele (cases 40, 41, 42, 44, 45 and 47; Figure 2B; Table S5). One HRD tumor carried a pathogenic variant in *BRCA2* with LOH of the wild-type allele (case 43; Figure 2B; Table S5), while *BRCA1* promoter hypermethylation was observed in another HRD tumor (case 49). No pathogenic variants in *BRCA1*, *BRCA2* or 13 additional HR-related genes, nor *BRCA1* promoter hypermethylation or large genomic rearrangements in *BRCA1*, were detected in the remaining HRD tumor (case 48) nor in the two HRI tumors (case 38 and 39). No pathogenic variants in *BRCA1* and/or *BRCA2*, or *BRCA1* promoter hypermethylation, were identified in any of the HRP tumors available for DNA analysis (Figure 2B).

### 2.4. Clinicopathological Characteristics

Characteristics of the 48 patients for which the functional HR status of the tumor could be established are shown in Table 1 and Table S6. The mean age of the patients was 62.5 (±1.7 SEM) years and 38/48 (79%) of the patients had been diagnosed with FIGO stage III or IV disease. The majority of patients were diagnosed with HGSOC 38/48 (79%). Tumor specimens were obtained from 33 ovarian tumors (i.e., solid) and from 16 ascites specimens. 

Twenty-four of the 48 (50%) patients underwent primary cytoreductive surgery followed by adjuvant platinum-based chemotherapy, while 21 of the 48 (44%) started on neoadjuvant platinum-based chemotherapy followed by interval cytoreductive surgery (Figure S4). In 39/48 (80%) patients, RECAP specimens were obtained at primary or interval cytoreductive surgery (i.e., primary disease) and in 10/48 (20%) patients at recurrent disease. A complete (i.e., no macroscopic rest tumor) or optimal cytoreduction (i.e., tumor rest < 1 cm in diameter) at initial surgery was achieved in 43/47 (92%) patients. One patient did not undergo interval cytoreductive surgery because of progressive disease after NACT (Table 1).

### 2.5. The RECAP Test as a Biomarker for Platinum-Based Therapy Response

Although this study was not primarily designed to assess the relationship between the RECAP score and clinical response to platinum-based therapy, a subgroup of patients was available that allowed exploration of the potential differences in platinum-based therapy response between patients with HRD and HRP tumors. Patients were included in this analysis if they met the following criteria: (1) RECAP classification of HRP or HRD, (2) the RECAP specimen was obtained at initial diagnosis of ovarian cancer (at primary or interval cytoreductive surgery), (3) the patient received platinum-based chemotherapy after the RECAP specimen was obtained, and (4) follow-up after first-line treatment was available (Figure S5). 

**Figure 3:**
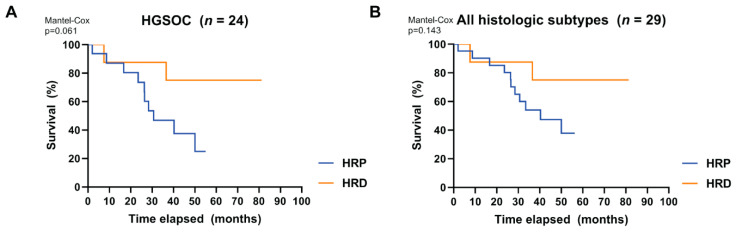
Overall survival of patients with high-grade serous ovarian carcinoma (HGSOC) only and diverse histologic subtypes. Kaplan–Meier estimates show the overall survival (%) over time (months) in a subgroup of patients whose tumors were analyzed by the RECAP test (see Materials and Methods). (**A**) Overall survival of 24 patients with HGSOC. (**B**) Overall survival of 29 patients with tumors of diverse histologic subtype. A Log-rank (Mantel-Cox) test was performed on both groups.

When considering the HGSOC patient group (Table 2), which included all of the HRD cases, we found a trend towards a better OS for patients with HRD tumors compared to patients with HRP tumors (Kaplan–Meier *p* = 0.061) (Figure 3A). When other histologic subtypes (all non-HRD) were included, this trend was weakened but maintained (Kaplan–Meier *p* = 0.143) (Figure 3B). 

However, patients with HRP or HRD tumors did not differ significantly in terms of best overall therapy response following first-line platinum-based chemotherapy (*p* = 1.000 HGSOC only; *p* = 0.483 all histologic subtypes). Median follow-up of the patients included in this analysis was 36.6 months. No difference was observed regarding DFS between patients with HRP and HRD tumors (*p* = 0.232 HGSOC only; *p* = 0.341 all histologic subtypes). 

## 3. Discussion

The development of a functional biomarker test that allows reliable identification of HRD ovarian malignancies is of broad oncologic interest, as it would facilitate the optimal selection of patients who are most likely to benefit from treatment with PARPi. Using an optimized RECAP test, we investigated the occurrence of HRD in 49 prospectively collected EOC. A substantial fraction of all EOCs in the current study consisted of HGSOC, and of these 26% were classified as HRD. The prevalence of HRD tumors amongst HGSOC that were related to *BRCA1/2* deficiency (including *BRCA1* promoter hypermethylation) was broadly in line with previous reports (Table S1) [17]. However, extended genetic analyses, including *RAD51C* promoter hypermethylation and mutation analysis in HR-related genes, suggest that the proportion of HRD in HGSOC may be as high as 50% [12,17,42], a finding also reported by others using either DNA signature-based approaches or a RAD51-based functional test [9,12,30,31]. Yet, using a similar functional HRD test, our group and Tumiati et al. observed a somewhat lower frequency of HRD (~30%) in HGSOC [35]. In our study, only one out of nine HRD tumors (11%) available for sequencing could not be explained by a *BRCA* defect, which is somewhat lower than expected based on previous studies [9,12]. As different methods have been used to identify HRD tumors, several factors may contribute to the observed differences in the prevalence of HRD tumors found in these studies. Firstly, patient cohorts differed in several aspects, such as the inclusion of platinum-sensitive primary and recurrent disease. Secondly, the “static” HRD status as measured by DNA-based approaches may overestimate the number of tumors that are “functionally” HRD because of, for example, undetected reversion mechanisms [16,38,39,40,41]. However, the RECAP test may possibly underestimate the frequency of HRD tumors, e.g., due to the inability to identify tumors that are unable to complete HR because formed RAD51 filaments are unstable [43]. 

The need for improved characterization of HR status in EOC has emerged in recent PARPi trials in which patient response was clearly related to HR status [4,5,9,12,44]. As expected, all trials reported significant benefits in EOC patients with underlying *BRCA1/2* defects (both germline and somatic) following PARPi treatment. Intriguingly, however, the PFS hazard ratios (HR) found in posthoc subanalysis clearly indicated that not only EOC patients with wt*BRCA* high HRD scores benefit from PARPi, but also a subset of EOC patients with wt*BRCA* tumors showing low HRD scores using DNA-based methods (MyChoice^®^ HRD test). Although unlikely based on available evidence, this outcome could be due to a PARPi response in otherwise HRP EOC. A more likely explanation is that current DNA-based assessment of HR status is not yet sufficiently accurate. Some of the HRD tumors might not be functional HRD and a number of functional HRD tumors might be missed when only DNA-based approaches are considered. 

Although, to date, no clinical trials have evaluated RAD51 foci formation as a biomarker for therapy response, several retrospective studies have reported that patients with HRD tumors (defined by a RAD51 functional test) show higher platinum sensitivity and improved survival rates [31,35]. Although our study was not designed or powered to reliably allow the predictive power of HRD as detected by the RECAP test to be assessed, we nevertheless found a trend towards better overall survival of HGSOC patients carrying HRD tumors compared to HRP tumors. Formally, we cannot rule out that this better overall survival is driven by the high frequency of *BRCA*-related HRD tumors (seven out of eight) for which improved therapy outcome has previously been reported. 

Additional studies will be required to determine whether the RECAP test is a reliable, comprehensive and efficient biomarker test for a PARPi response. Ideally, these studies should include a comprehensive comparison of the performance of various HRD biomarker tests with respect to sensitivity, specificity, take-rate and costs.

The RECAP test as described here has many advantages over DNA-based tests in terms of cost, speed of turnaround and simplicity of analysis once implemented. The cost per sample for the RECAP test is more than 10-fold lower than the costs of running a *BRCA* NGS gene panel (Table S3). In addition, the RECAP test can be completed in less time than a *BRCA* NGS gene panel (Table S4). Another major strength of the RECAP test is that it allows identification of HRD tumors independently of *BRCA* status, while correctly assessing *BRCA-*related tumors with reversion mutations as being HRP [36,37,41], in contrast to DNA-based tests [16,38,39,40]. In our study, the RECAP test identified all tumors with *BRCA* defects as HRD, further underlining the high reliability of the test. As some laboratories nowadays offer *BRCA* tumor testing to identify patients for follow- up germline *BRCA* testing, the use of the RECAP test as a prescreen for *BRCA* testing would substantially reduce the number of tumors to be sequenced to identify these patients. 

The RECAP test is not without its limitations. It relies on fresh or cryopreserved tumor specimens and requires induction of DSBs by ionizing radiation or chemical compounds such as platinum-based compounds or PARPi [29,30,32,33,36,37]. The percentage of samples that did not pass our stringent quality control (29%) is higher than reported for DNA-based analyses (15%) [4,9]. 

Initial set-up and implementation in a routine diagnostic setting might therefore be challenging in some laboratories. Recently, an attractive adaptation of the RECAP procedure was proposed that maintains the advantages of the test while avoiding the drawbacks. This new approach is innovative because it now allows assessment of RAD51 foci in FFPE breast tumor samples directly [45,46]. We now propose to establish whether the use of FFPE material can serve as a reliable substitute for fresh tumor tissue, comparing fresh tumor specimens and matching archival FFPE tumor blocks on RAD51 scores, ideally in large cohorts of different tumor types. Should the FFPE-based method prove to be as reliable as the current RECAP test, the use of RAD51 as a biomarker for the identification of HRD tumors will become feasible in many more diagnostic laboratories, facilitating the rapid and reliable identification and selection of patients who may derive the most benefit from PARPi treatment.

In conclusion, we show that functional analysis of HR status in EOC by the RECAP test enables fast and reliable identification of tumors with a deficiency in HR. The RECAP test is therefore an attractive alternative to DNA-based HRD tests and warrants further development as a clinical grade test.

## 4. Materials and Methods

### 4.1. EOC Patient Material

Fresh tumor tissue or ascites fluid from patients with primary or recurrent EOC who underwent cytoreductive surgery or drainage of ascites fluid at Leiden University Medical Center (LUMC) was prospectively collected if sufficient material was available for research. In total, 70 specimens (43 solid tumors and 27 ascites) were obtained from 66 patients between June 2010 and July 2017.

After surgical removal of the tumor, macroscopic dissection was performed for diagnostic purposes at the Department of Pathology. When available, a tumor tissue fragment (minimum of 0.5–1 cm^3^) was transferred to OSE medium (Wisent Bioproducts, cat. 316-030-CL) supplemented with 10% Fetal Bovine Serum (FBS) (Bodinco), 1% penicillin (100 U/mL) and streptomycin (0.1 mg/mL), kept at 4 °C and processed within 24 h of surgical resection (Figure 1A). Ascites was collected in fluid drainage bags, kept at 4 °C and used within 24 h after fluid drainage. Surplus tumor specimens were cryopreserved to enable comparison of test outcomes in fresh versus cryopreserved specimens (see below).

All specimens were coded with a unique research code. The study protocol has been approved by the Medical Ethics Committee of the LUMC on 7 February 2011 and 24 May 2017 (P10.226 and G17.041) and specimens were handled according to the Code for Proper Secondary Use of Human Tissue in the Netherlands as established by the Dutch Federation of Medical Scientific Societies. 

### 4.2. Cryopreservation and Thawing of Tumor Specimens 

Surplus tumor specimens were cryopreserved in ampules containing 500 µL Recovery Cell Culture Freezing Medium (Gibco, cat. 12648010). Cells isolated from ascites were collected and cryopreserved at approximately 10 million cells per ampule containing 500 µL of Recovery Cell Culture Medium. Ampules were placed in a freezing container (Nalgene Mr. Frosty, Sigma, cat. C1562) overnight at −80 °C before transfer to liquid nitrogen storage. Cryopreserved tumor specimens were thawed quickly in a 37 °C water bath. Solid tumor specimens were washed in pre-warmed (37 °C) OSE medium containing 40% FBS by gentle rotation of the tube for 5 min and subsequently transferred to 2.5 mL OSE medium supplemented with 10% FBS and pen/strep, and next incubated on a 60 rpm rotation platform at 37 °C in a 5% CO_2_ humidified atmosphere overnight prior to irradiation. After thawing, cells from ascites were collected by centrifugation (1000 rpm, 5 min), washed for 5 min in the presence of pre-warmed (37 °C) OSE medium containing 40% FBS, collected by centrifugation and supplemented in culture medium for subsequent use (see below).

### 4.3. REcombination CAPacity (RECAP) Test 

The RECAP test was performed according to our previously published method [37], but was adapted for ascites specimens as follows: Tumor cells were obtained by centrifugation (5 min, 1000 rpm) of one liter of ascites fluid, cell pellets were then washed in lysis buffer (155 mM NH_4_CL/21 mM Tris) to remove red blood cells, and after a second round of centrifugation, cell pellets were washed in OSE medium supplemented with 10% FBS and penicillin-streptomycin (pen/strep). Approximately ten million cells were seeded in 20 mL OSE medium supplemented with 10% FBS and pen/strep prior to incubation and irradiation. After fixation, cells were centrifuged (1000 rpm, 5 min), washed in 70% ethanol and embedded in Shandon Cytoblock gel (Thermo Scientific, Waltham, MA USA, cat. 7401151). In addition, all tumor specimens were subjected to quality assessment. 

### 4.4. Quality Assessment

To determine whether the tissue quality and the number of tumor cells was sufficient for analysis in the RECAP test, three quality assessments were applied as summarized in Figure 1B and Figure S2A. 

First, the quality of the tumor specimen was assessed by a pathologist (TB) using a hematoxylin and eosin (H&E) stained section of the irradiated tumor used in the RECAP test (quality control 1, QC1). The tissue quality (1–2 = poor, 3–4 = moderate and 5–6 = good) was determined on the basis of the sum of the tissue vitality (1 = poor, 2 = moderate and 3 = good) and tumor percentage (0 = <5%, 1 = 5–20%, 2 = 21–50% and 3 = >50%). When a tumor specimen had a total tissue quality score of ≤2, cryopreserved tissue was thawed, and the procedure was repeated. If the total tissue quality score of this sample was also ≤2, the specimen was excluded from further analysis (Table S4). 

To aid in the identification of tumor cells in ascites specimens, we included a p53 immunostaining as a second quality control (QC2) since over 98% of HGSOC have been reported to be *TP53*-mutant [47]. Immunohistochemistry staining for p53 (Agilent Dako, cat. M7001) was performed as described previously [48]. A pathologist (TB) used a combination of H&E and p53-IHC stained slides to either include (sufficient cancer cells available) or exclude (insufficient cancer cells available) ascites cases from the study (Table S4). 

Third, yH2AX immunostaining was included as a proxy for the presence of DNA DSBs (quality control 3, QC3). Immunohistochemistry was performed as described previously [48]. The primary yH2AX antibody (mouse, monoclonal, clone JBW301, Sigma-Aldrich, Zwijndrecht, The Netherlands, cat. 05-636) was diluted 1:40,000 in block buffer and incubated at room temperature (RT) o/n. The secondary antibody (BrightVision poly-HRP-anti-Mouse, Immunologic, Duiven, The Netherlands, cat. VWRDPVVM0110HRP) was incubated for one hour at RT according to the manufacturers’ protocol. A pathologist (TB) scored the slides based on the presence (inclusion of the sample) or absence (exclusion of the sample) of yH2AX foci.

A total of 70 EOC specimens (43 solid tumors and 27 ascites specimens) (Figure S2A) were collected from 66 patients. Among these tumor specimens 47/70 passed quality control according to QC1 (Materials and Methods, 30 solid and 17 ascites). Of the fresh specimens that failed QC1, 11 (3 solid and 8 ascites) could still be included by using cryopreserved material (Figure S1), resulting in a total of 58/70 (33 solid and 25 ascites) tumor specimens that passed QC1. Seventeen of the 25 ascites specimens passed QC2 (Materials and Methods, p53 staining). All tumor specimens were positive for yH2AX foci and therefore passed QC3.

### 4.5. Immunofluorescence Staining for RAD51 and Geminin

Immunofluorescence staining for RAD51 (RAD51 (mouse, monoclonal, GeneTex, Alton Pkwy Irvine, CA USA, cat. GTC70230)) and geminin (rabbit, polyclonal, ProteinTech, Manchester, United Kingdom, cat. 10802-1-AP)) was performed as described previously [37], with the following modifications: Tissue sections were incubated at 60 °C overnight on SuperFrost Plus microscope slides (75 × 25 mm, VWR, Amsterdam, The Netherlands, cat. 631-0108) prior to immunofluorescence staining and no EdU immunostaining was performed (Figure S3). 

### 4.6. Scoring of RECAP Tumor Specimens

The scoring of RECAP tumor specimens was blinded for genetic data (*BRCA* status). Tissue sections stained for DAPI, geminin (G2/S phase marker) and RAD51 were manually scored using a Zeiss Axio Imager D2 microscope with a HXP 120C light source. DAPI was used to localize tumor cells in the tissue section based on morphology. Cells were considered geminin-positive (GMN^+^) if the nucleus was homogenously stained. GMN^+^ cells were considered RAD51^+^ if there were at least 5 nuclear foci visible. A minimum of 40 GMN^+^ cells, randomly chosen in vital tumor areas (defined by the lack of necrosis visible with DAPI) were scored. Specimens with less than 40 GMN^+^ cells were excluded from the analysis (Figure 1C and Figure S2B). 

The RECAP score is the average percentage score, for two independent observers, of GMN+ cells with RAD51 foci. Tumors were allocated to one of three groups: HR-Proficient (HRP; 51–100%), HR-Intermediate (HRI; 21–50%) or HR-Deficient (HRD; 0–20%). The two hour post-irradiation incubation time point and the thresholds for HR status assignment were determined in a previous study on breast tumors [37]. In this and subsequent studies on breast and endometrial carcinomas these settings allowed unequivocal discrimination between *BRCA* wildtype and *BRCA1/2* deficient tumors (including those with promoter hypermethylation of *BRCA1*) while identifying an additional group of HRD tumors not related to *BRCA1/2* [36,37]. In EOC, with these settings RAD51 foci formation was observed in the majority of replicating tumor cells in *BRCA* wildtype tumors while correctly identifying *BRCA1/2* deficient tumors. The Cohen kappa coefficient (k) was used to measure interobserver and intertest agreement.

### 4.7. Tumor DNA Isolation

Tumor DNA was isolated from formalin-fixed paraffin-embedded (FFPE) tissue blocks either by taking three 0.6 mm tumor cores or by microdissection of tumor areas with at least 70% tumor cells (10 mm slides). Fully automated DNA isolation was performed using the Tissue Preparation System (Siemens Healthcare Diagnostics) as described previously [37]. An H&E slide (5 µm) was prepared for each FFPE tissue block to determine tumor percentage prior to tumor DNA isolation. The Qubit dsDNA HS Assay Kit was used for DNA quantification according to manufacturer’s protocol (Qubit 2.0 Fluorometer, Invitrogen, Waltham, MA USA, cat. Q32851). 

### 4.8. Next-Generation Sequencing

Next-generation sequencing (NGS) was performed using 40 ng of tumor DNA per sample isolated from FFPE tissue blocks. The mean tumor cell percentage of included samples was 62% (range: 10–90%). All tumors (HRD, HRI, and HRP) have been sequenced for *BRCA1* and *BRCA2* and analyzed for promoter hypermethylation of *BRCA1*. The non-*BRCA* HRD tumor and the two HRI tumors were subsequently analyzed for pathogenic variants in 13 additional HR-related genes and large genomic rearrangements in *BRCA1*. 

The custom Ampliseq HDR15v1-panel (Thermo Fisher) was used for variant detection in the coding exons of the following HR-associated genes: *ATM* (not covered by design: exon 25 (1225–1231), exon 36 (1813–1821), exon 52 (2576–2596)), *BARD1*, *BRCA1*, *BRCA2*, *BRIP1*, *CDK12* (not covered by design: exon 1 (294–302)), *CHEK1*, *CHEK2*, *FANCL*, *PALB2*, *PPP2R2A*, *RAD51B*, *RAD51C* (not covered by performance, exon 5 (236–241)), *RAD51D* (not covered by performance, exon 5 (130–160)), and *RAD54L*. Details on request (TVW, NS). Mutation and LOH analysis of the NGS data was performed as described previously by de Jonge et al. [49]. Variants were categorized using the 5-tier pathogenicity classification according to Plon et al.: Class 1 = benign, Class 2 = likely benign, Class 3 = variant of unknown significance (VUS), Class 4 = likely pathogenic, and Class 5 = pathogenic [50].

### 4.9. BRCA1 Promoter Hypermethylation by MS-MLPA 

Analysis of *BRCA1* promoter hypermethylation by MS-MLPA was performed as described previously [37].

### 4.10. BRCA1 MLPA 

Copy number variant (CNV)-MLPA was performed as described previously [49].

### 4.11. Clinical Response Evaluation and Follow-Up

Clinical follow-up data were retrospectively collected for all patients whose tumor specimens had an informative RECAP score. Ovarian cancer staging was performed according to the 2014 International Federation of Gynecology and Obstetrics (FIGO) guidelines [51]. The study was conducted in accordance with the Declaration of Helsinki and the Dutch Code of Good Conduct. 

Primary therapy response after first-line treatment and overall therapy response at the last check-up were assessed by physical and gynecological examination, measurement of tumor marker CA125 and/or computed tomography (CT) scans according to the RECIST version 1.1 guideline [52]. In patients with CA125 levels < 35 KU/L [53] and no clinical signs of tumor by physical and gynecological investigation, a standard CT scan was not always performed after first-line treatment (Figure S4). Primary therapy response after first-line treatment was noted as a complete or partial response, or as stable or progressive disease according to the WHO criteria. The best overall therapy response is defined as the best recorded response from the start of the treatment to disease progression/recurrence.

Disease-Free Survival (DFS) was defined as the period between start of treatment, i.e., primary cytoreductive surgery, staging procedure or start of platinum-based chemotherapy, and the first observation of recurrent or progressive disease or death due to any cause, whichever occurred first. A patient was considered platinum-sensitive when no recurrence or progression was noted for ≥6 months after the last chemotherapy. When recurrence or progression occurred within <6 months after the last chemotherapy, a patient was considered platinum-resistant. Overall Survival (OS) was determined from the date of start of treatment i.e., date of primary surgery or start of platinum-based chemotherapy to the date of death from any cause. Follow-up time was calculated from the date of start of treatment until the last check-up before cut-off for the final analysis or the date of death from any cause (Figure S4). 

### 4.12. Statistical Analysis

Statistical analysis of clinical data was performed with SigmaStat 3.5 (Systat Software Inc, San Jose, CA, USA) and Graphpad Prism 8.0 (Graphpad Software, San Diego, CA, USA). Student’s *t*-tests were performed on numerical data when normality criteria were met, otherwise Mann-Whitney U tests were performed. Chi-square tests were performed on categorical data when normality criteria were met and Fisher’s exact tests were performed when data were not normally distributed. Kaplan–Meier plots of follow-up were generated in Graphpad Prism 8.0 for OS. Images were produced with GraphPad Prism 8.0 and Adobe Creative Suite CS6 (Adobe, San Jose, CA, USA).

## 5. Conclusions

In this manuscript we describe the use of the REcombination CAPacity (RECAP) test to identify HRD ovarian carcinomas. We found that all HRD ovarian carcinomas in our cohort were of the high-grade serous (HGSOC) histologic subtype. The RECAP test showed that 26% (10/39) of HGSOCs were HRD. Of the HRD tumors available for sequencing, 8/9 showed pathogenic *BRCA1/2* variants or *BRCA1* promoter hypermethylation, indicating that the RECAP test matches and exceeds the detection capacity of DNA-based tests, but more rapidly and at lower costs. Furthermore, we found a trend towards better overall survival of HGSOC patients with HRD tumors compared to patients with HR proficient tumors. Overall, we show that the RECAP test is rapid, reliable, and as such, a good alternative to DNA-based tests.

## Figures and Tables

**Table 1:** Clinicopathological characteristics stratified for homologous recombination capacity of the tumor.

Characteristics		Total Cohort *n* = 48	HRP *n* = 36	HRI *n* = 2	HRD *n* = 10
Age at diagnosis	Mean (±SEM)	62.5 (±1.7)	63.6 (±1.9)	76.5 (±9.2)	55.9 (±3.2)
FIGO stage	I (I; IA; IC)	4 (8.3%)	3 (8.3%)		1 (10%)
	IIB	6 (12.5%)	6 (16.7%)		
	III (IIIA; IIIC)	31 (64.6%)	22 (61.1%)	2 (100%)	7 (70%)
	IV	7 (14.6%)	5 (13.9%)		2 (20%)
Histologic subtype	High-Grade Serous	38 (79.2%)	26 (72.2%)	2 (100%)	10 (100%)
	Low-Grade Serous	3 (6.3%)	3 (8.3%)		
	Endometrioid	4 (8.3%)	4 (11.1%)		
	Clear cell	1 (2.0%)	1 (2.8%)		
	Mucinous	2 (4.2%)	2 (5.6%)		
Tumor specimen type *	Solid tumor	33 (67.3%)	24 (66.7%)	2 (100%)	7 (70%)
	Ascites	16 (32.7%)	13 (36.1%)		3 (30%)
Tumor specimen obtained *	Primary disease	39 (79.6%)	28 (75.7%)	2 (100%)	9 (90%)
	Recurrent disease	10 (20.4%)	9 (24.3%)		1 (10%)
Primary treatment strategy	Staging	3 (6.3%)	3 (8.3%)		
	Primary cytoreductive surgery	24 (50%)	18 (50%)	1 (50%)	5 (50%)
	Neoadjuvant chemotherapy	21 (43.7%)	15 (41.7%)	1 (50%)	5 (50%)
Residual tumor after cytoreductive surgery **	Complete (0 cm)	25 (53.2%)	16 (45.7%)	2 (100%)	7 (70%)
	Optimal (<1 cm)	18 (38.3%)	17 (48.6%)		1 (10%)
	Not optimal (>1 cm)	4 (8.5%)	2 (5.7%)		2 (20%)
Previous cancer(s)	Ovarian and/or breast	15 (31.2%)	10 (27.8%)		4 (40%)
	None	33 (68.8%)	26 (72.2%)	1 (50%)	6 (60%)

* Two tumor specimens were derived from one patient. ** One patient with an HRP tumor did not undergo cytoreductive surgery. Abbreviations: HRP = HR-proficient; HRI = HR-Intermediate; HRD = HR-Deficient; FIGO = International Federation of Gynecology and Obstetrics.

**Table 2:** Correlation between RECAP score and clinical response.

Clinical Parameters		Diverse Histologic Subtypes			HGSOC		
		HRP *n* = 21	HRD *n* = 8	HRP vs. HRD	HRP *n* = 16	HRD *n* = 8	HRP vs. HRD
Best overall therapy response— as measured with CT scans/CA125 levels	Complete Response (CR)	17 (81.0%)	7 (87.5%)	*p* = 0.483	12 (75.0%)	7 (87.5%)	*p* = 1.000
	Partial Response (PR)	3 (14.3%)			3 (18.8%)		
	Progressive Disease (PD)	1 (4.7%)	1 (12.5%)		1 (6.2%)	1 (12.5%)	
Disease Free Survival (DFS)—Time between start of treatment and progressive disease	Months (median)	16.0	23.8	*p* = 0.341	15.4	23.8	*p* = 0.232
Overall therapy response last check-up—as measured with CT scans/CA125 levels	Complete Response (CR)	6 (28.6%)	5 (62.5%)	*p* = 0.092	3 (18.8%)	5 (62.5%)	*p* = 0.058
	Partial Response (PR)	1 (4.7%)	1 (12.5%)		1 (6.2%)	1 (12.5%)	
	Stable Disease (SD)	2 (9.5%)			2 (12.5%)		
	Progressive Disease (PD)	12 (57.2%)	2 (25.0%)		10 (62.5%)	2 (25.0%)	
Overall Survival (OS)	Yes	10 (47.6%)	6 (75%)	*p* = 0.238	6 (37.5%)	6 (75%)	*p* = 0.193
	No	11 (52.4%)	2 (25%)		10 (62.5%)	2 (25%)	

DFS and follow-up time were tested with the Mann-Whitney U test. Other clinical parameters were tested with Fisher’s exact test. Statistically significant p-value in bold. Abbreviations: HGSOC = High-grade serous ovarian carcinoma; HRP = HR-Proficient; HRD = HR-Deficient; CT = computed tomography.

## Supplementary Materials

The following are available online at https://www.mdpi.com/2072-6694/12/10/2805/s1, Figure S1: Validation of the RECAP test using fresh and cryopreserved tumor specimens, Figure S2: Flowchart illustrating inclusion criteria for RECAP specimens and take-rate, Figure S3: Microscopy illustration of HRP and HRD solid and ascites ovarian tumor specimens classified by the RECAP test, Figure S4: Timeline treatment procedure EOC patients, Figure S5: Flowchart illustrating patient inclusion for comparison of RECAP scores with therapy response, Table S1: Identification of HR-deficient tumors by functional and genomic analyses, Table S2: Estimated workload of *BRCA* next-generation sequencing gene panel testing versus the RECAP test, Table S3: Estimated cost prices for a *BRCA* next-generation sequencing gene panel versus the RECAP test, Table S4: Excluded tumor specimens based on tissue quality, *p53* status or the RECAP test, Table S5: Pathogenic variants in *BRCA1* and *BRCA2* identified in HR-deficient ovarian carcinomas and Table S6: Extended clinicopathological characteristics of the ovarian cohort stratified for homologous recombination repair capacity as determined by the RECAP test.
