# Supplementary material for: The RECAP Test Rapidly and Reliably Identifies Homologous Recombination-Deficient Ovarian Carcinomas
Source: Cancers (Basel). 2020 Sep 29;12(10):2805. doi: 10.3390/cancers12102805 (PMC7650677; doi:10.3390/cancers12102805)
Supplement: Supplementary file 1 [file cancers-12-02805-s001.zip › cancers-930804-supplementary.pdf]

cancers

Supplementary Materials: The RECAP Test Rapidly and Reliably Identifies Homologous Recombination-Deficient Ovarian Carcinomas

Lise M. van Wijk, Sylvia Vermeulen, Matty Meijers, Manuela F. van Diest, Natalja T. ter Haar, Marthe M. de Jonge, Nienke Solleveld-Westerink, Tom van Wezel, Dik C. van Gent, Judith R. Kroep, Tjalling Bosse, Katja N. Gaarenstroom, Harry Vrieling and Maaike P.G. Vreeswijk

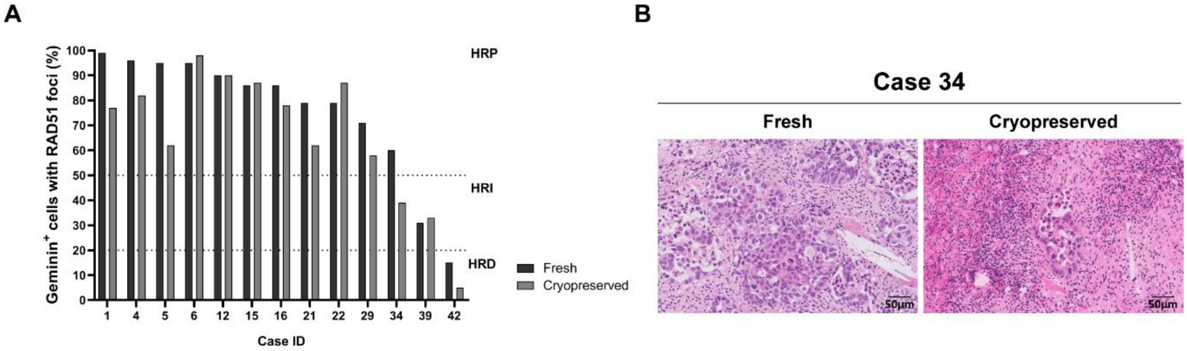

Figure S1. Validation of the RECAP test using fresh and cryopreserved tumor specimens. Case IDs correspond to case IDs described in Figure 2, Supplementary Figure S3, Supplementary Table S5 and Supplementary Table S6. **A**) Thirteen tumor specimens were selected to assess whether cryopreserved tumor tissue can substitute for fresh tumor tissue when determining HR status using the RECAP test. All thirteen cases (matched fresh and cryopreserved) passed the quality assessment, and the HR group assignment based on the RECAP score was highly concordant between fresh and cryopreserved tumor specimens (12/13). RECAP scores for cryopreserved tumor specimens displayed a median score difference of 9% (range: 2% - 24%) between two independent observers, with a high interrogator reliability for final HR group assignment (κ = 1). **B**) Only one specimen was assigned to another HR group, i.e. case 34 was categorized as HRI using cryopreserved tissue (RECAP score 39%), but as HRP using the fresh specimen (RECAP score 60%). This discrepancy may have been caused by tumor heterogeneity, as multiple tumor fields were visible in the fresh specimen (left) but only one tumor field was present in the cryopreserved specimen (right), as visualized in the H&E slides. Abbreviations: HRP = HR-Proficient; HRI = HR-Intermediate; HRD = HR-Deficient; GMN^{+} = geminin-positive; H&E = Hematoxylin and Eosin.

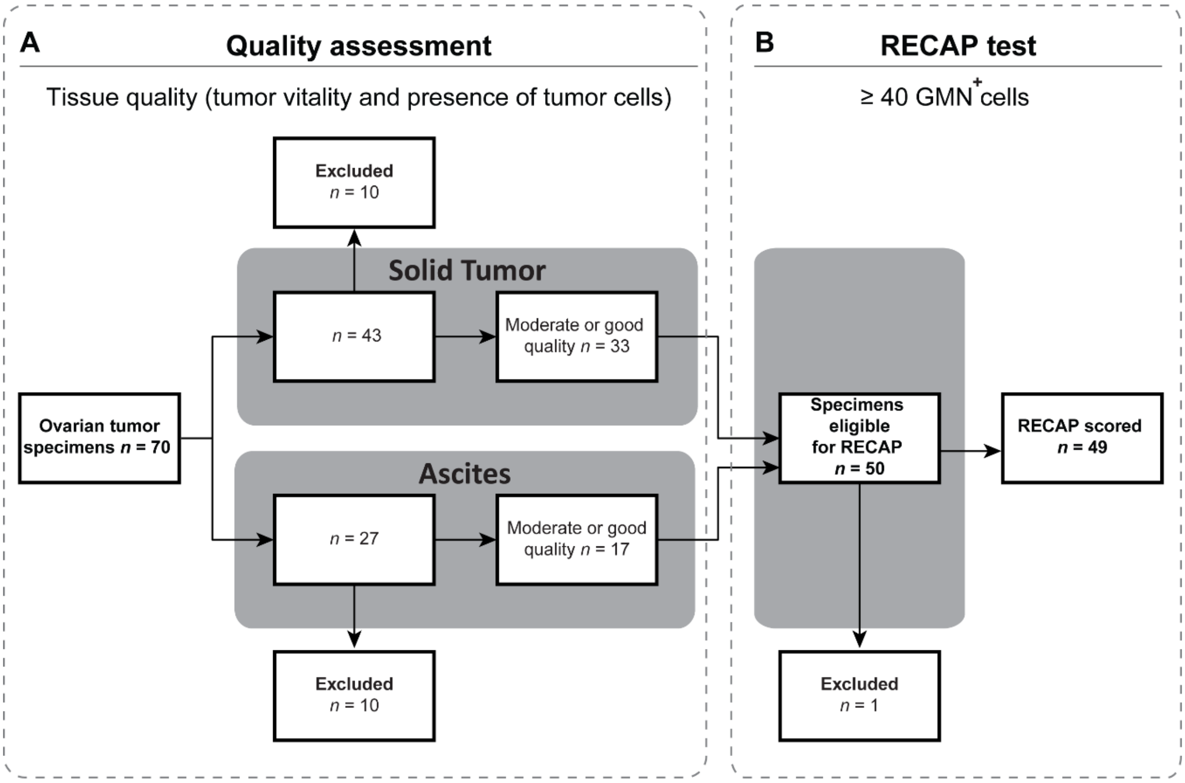

Figure S2. Flowchart illustrating inclusion criteria for RECAP specimens and take-rate. A) Quality assessment was performed as described in Figure 1 and Materials & Methods. Ten solid tumors were excluded due to poor tissue quality. Ascites specimens were excluded due to poor tissue quality (n = 2) and lack of tumor cells (n = 8). **B)** One ascites specimen was excluded. In total, 49 tumor specimens were scored by the RECAP test. Abbreviations: RECAP = REcombination CAPacity; GMN = geminin-positive.

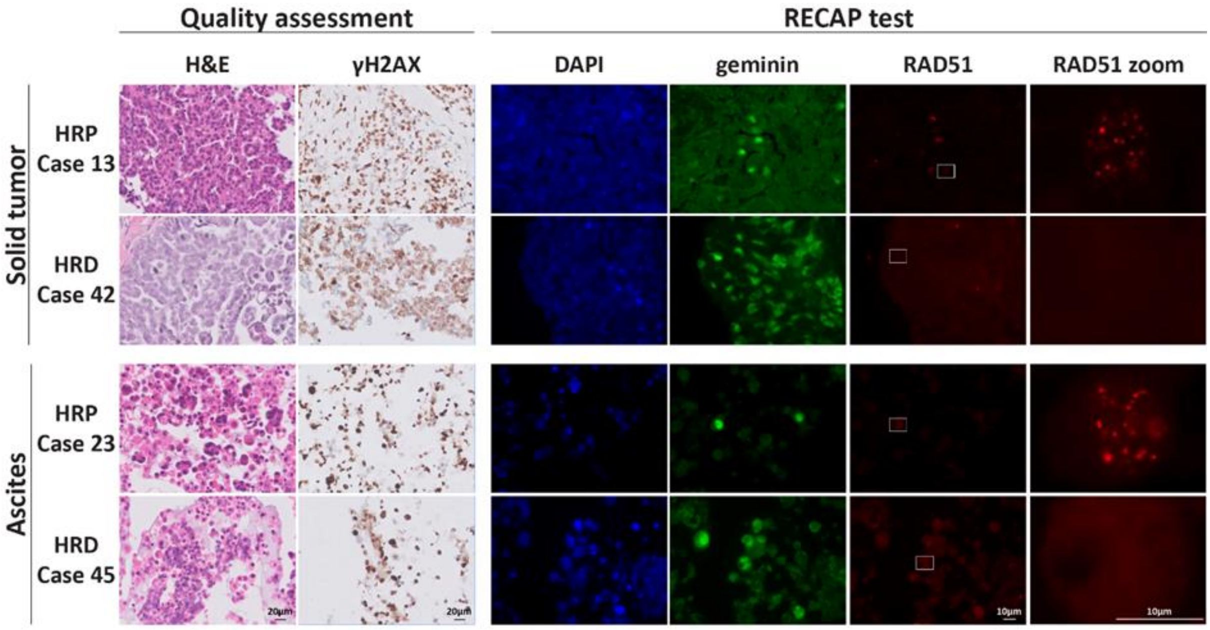

Figure S3. Microscopy illustration of HRP and HRD solid and ascites ovarian tumor specimens classified by the RECAP test. Tumor tissue quality (H&E) was approved by an experienced pathologist and the presence of DNA DSBs was confirmed by γH2AX immunohistochemistry. DAPI was used to identify tumor cells based on morphology. Geminin, a G2/S phase marker, was used to identify cells in G2/S phase. The RAD51 zoom images represent enlargements of the cells surrounded by white boxes in the RAD51 column. Cases correspond with cases described in Figure 2, Supplementary Figure S1, Supplementary Table S5 and Supplementary Table S6. Abbreviations: HRP = HR-Proficient; HRD = HR-Deficient; RECAP= REcombination CAPacity; H&E = Hematoxylin and Eosin.

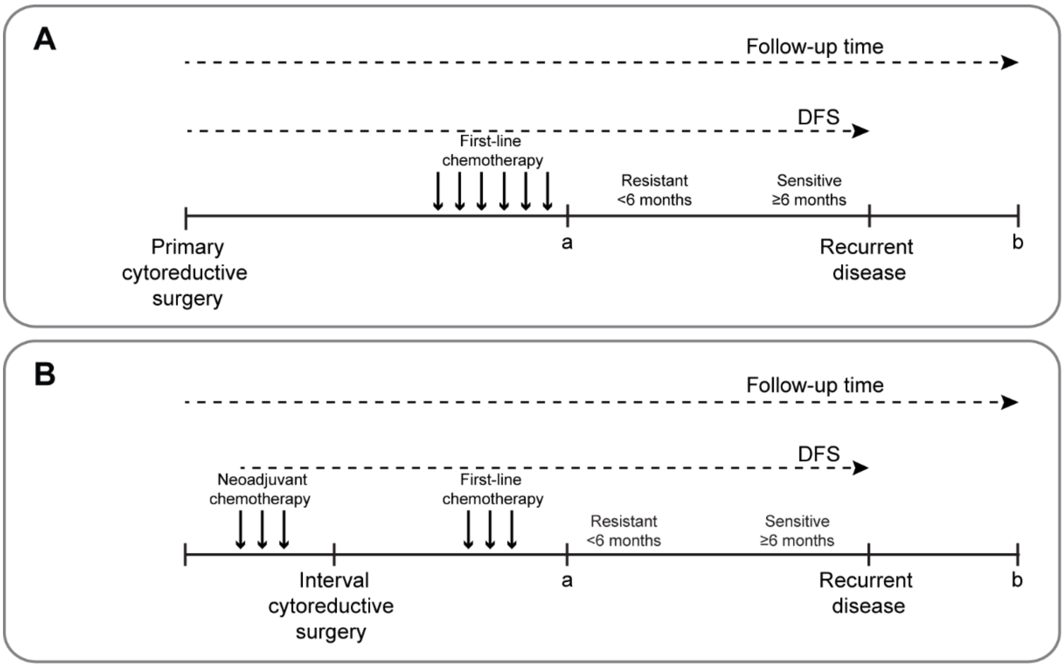

Figure S4. Timeline treatment procedure EOC patients. A) Treatment procedure for EOC patients undergoing primary cytoreductive surgery combined with first-line chemotherapy. **B)** Treatment procedure for EOC patients undergoing neoadjuvant chemotherapy combined with interval cytoreductive surgery and first-line chemotherapy. RECAP tumor specimens were obtained during the primary cytoreductive surgery, interval cytoreductive surgery or during recurrent disease. Clinical treatment outcome (indicated with 'a' and 'b') was measured directly after first-line chemotherapy treatment (a = best overall therapy response) and at the last check-up or death (b = OS). The follow-up time was measured as the time between the start of treatment and the last check-up or death. DFS was measured as the time between the start of treatment and recurrence/progression of disease. A patient is considered platinum-sensitive when no recurrence or progression occurs for ≥6 months after the last chemotherapy. When recurrence or progression occurs in <6 months after the last chemotherapy, a patient is considered platinum-resistant. Abbreviations: EOC = epithelial ovarian carcinoma; DFS = Disease Free Survival; OS = Overall Survival.

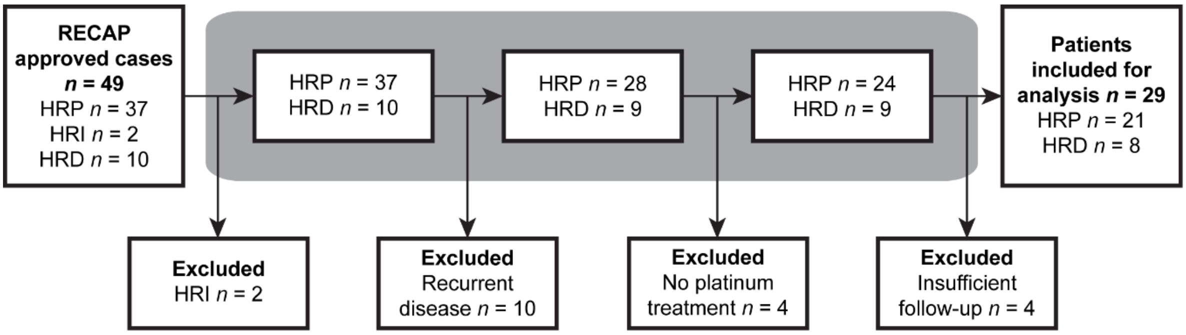

Figure S5. Flowchart illustrating patient inclusion for comparison of RECAP scores with therapy response. To determine if the RECAP score was predictive for the clinical response to platinum treatment, patients were included in the analysis based on the following criteria: i) the classification based on the RECAP score was HR-proficient or HR-deficient, ii) the tumor specimen was obtained during primary disease (primary or interval surgery), iii) the patient received platinum-based chemotherapy and iv) complete follow-up data after first-line chemotherapy was available. Abbreviations: HR = Homologous Recombination; HRP = HR-Proficient; HRI = HR-intermediate; HRD = HR-Deficient; RECAP = REcombination CAPacity.

© 2020 by the authors. Licensee MDPI, Basel, Switzerland. This article is an open access article distributed under the terms and conditions of the Creative Commons Attribution (CC BY) license (http://creativecommons.org/licenses/by/4.0/).
